# Intracellular Spatial Localization Regulated by the Microtubule Network

**DOI:** 10.1371/journal.pone.0034919

**Published:** 2012-04-19

**Authors:** Jing Chen, Jennifer Lippincott-Schwartz, Jian Liu

**Affiliations:** 1 National Heart, Lung and Blood Institute, National Institutes of Health, Bethesda, Maryland, United States of America; 2 National Institute of Child Health and Human Development, National Institutes of Health, Bethesda, Maryland, United States of America; Stockholm University, Sweden

## Abstract

The commonly recognized mechanisms for spatial regulation inside the cell are membrane-bounded compartmentalization and biochemical association with subcellular organelles. We use computational modeling to investigate another spatial regulation mechanism mediated by the microtubule network in the cell. Our results demonstrate that the mitotic spindle can impose strong sequestration and concentration effects on molecules with binding affinity for microtubules, especially dynein-directed cargoes. The model can recapitulate the essence of three experimental observations on distinct microtubule network morphologies: the sequestration of germ plasm components by the mitotic spindles in the *Drosophila* syncytial embryo, the asymmetric cell division initiated by the time delay in centrosome maturation in the *Drosophila* neuroblast, and the diffusional block between neighboring energids in the *Drosophila* syncytial embryo. Our model thus suggests that the cell cycle-dependent changes in the microtubule network are critical for achieving different spatial regulation effects. The microtubule network provides a spatially extensive docking platform for molecules and gives rise to a “structured cytoplasm”, in contrast to a free and fluid environment.

## Introduction

The microtubule network is commonly recognized as the major mechanical skeleton that drives cell division. Numerous seminal experimental observations led us to speculate that the microtubule network could also serve as a spatial regulator for cellular components. Many molecules can bind with microtubules either directly, or indirectly through other microtubule-binding molecules. Binding to microtubules causes the partial sequestration of the molecules by the microtubule network, the degree of which depends on the binding affinities, as well as the microtubule density. In addition, motor protein-mediated binding leads to convective fluxes that help rearrange the spatial localization of the cargo molecules. For example, in pace with the progression of mitosis, a number of mitotic spindle checkpoint proteins accumulate at the poles of the mitotic spindle via the active transport of dyneins along the microtubules [1], [2]. Microtubule-mediated spatial regulation also plays critical functional roles in various biological processes, e.g. the determination of embryo polarity and cell fates in the syncytial *Drosophila* embryo [3], [4], the establishment of dorsal-ventral axis in the *Xenopus* embryo [5], [6], the asymmetric cell division in the *Drosophila* central brain neuroblast [7], [8], [9], [10], [11].

The effectiveness of microtubule-mediated sequestration and its consequent spatial regulation can be much higher than intuitively expected. For example, in the *Drosophila* syncytial embryo, the germ plasm components translocate in a dynein-dependent manner from the posterior embryo cortex onto the mitotic spindles which have migrated to the vicinity [4]. The germ plasm components become strongly concentrated at the poles of these spindles. The transport process is almost leak-free: the germ plasm components only concentrate at one pole of the spindle if the spindle happens to orient somewhat perpendicular to the cortex, keeping the distal pole a few micrometers away from the cortex (cf. fig. 6B of [4]). The leak-free behavior is surprising, because dyneins travel along the microtubule processively only for ∼1 µm [12]. During the journey of 5∼10 µm towards the spindle pole, they almost certainly fall off the microtubule and are then supposed to rapidly diffuse into the cytoplasm. Unless the dyneins rebind to microtubules very soon, the contrast in concentrations cannot emerge between the two poles.

In this work we use computational models to study the spatial regulation by the microtubule network. We first show that the microtubule density in a mitotic spindle is high enough to cause the biased concentration of dyneins and their cargoes onto one pole, as observed by Lerit *et al* [4]. We then extend the results and propose a possible mechanism for the asymmetric distribution of cell fate determinants during the asymmetric cell division. Finally, we show that the partial sequestration under modest microtubule densities explains the delayed diffusion and the photobleach recovery pattern of Dorsal proteins in the syncytial *Drosophila* embryo [13]. Our work suggests that the microtubule network, by dynamically altering its own architecture, is able to achieve different spatial sequestration effects and facilitate different cellular processes at various stages of the cell cycle.

## Results

### Agent based model

We used agent-based stochastic simulation to compute the spatial distribution of the molecules with binding affinity for microtubules. We traced the spatial trajectory of each molecule. They diffuse in the cytoplasm when unbound. And they travel along the microtubule when bound, the velocity of which depends on the associated motor proteins, and is zero if not associated with a motor.

The microtubule-binding molecule or motor-cargo complex is simplified as a spherical particle of 10 nm in diameter. In reality, these particles can assume various shapes, and the dimensions can differ up to an order of magnitude. But the size and shape does not significantly affect the binding dynamics as long as the binding is diffusion-limited (Figure S1, increase of particle radius by an order of magnitude only doubles the effective binding rate). A free particle diffuses in the cell with a diffusion coefficient, *D* (1∼20 µm^2^/s). It binds to a microtubule when the two objects are close by within a critical distance, *d_0_* ( = 0.8 nm). It unbinds from the microtubule with a dissociation rate, *k* (∼1 s^−1^). If the particle represents a molecular motor and/or motor-cargo complex, then the particle, when bound, will travel along the microtubule unidirectionally with a velocity, *V* (∼1 µm/s). The parameters of the model are taken from independent experimental measurements/estimates, with references and/or reasoning listed in Table 1.

**Table 1:** Parameters used to simulate the dynein-mediated transport by microtubule spindle.

Parameter	Meaning	Used Value	Source/Reason
Δ*t*	Time step for agent-based simulation	10^−4^ s	Less than the time scale of rotational diffusion of dynein (see **Information S1**)
*N_s_*	Number of spindle microtubules from each pole	800	(∼40) kinetochores×(∼20) microtubules per kinetochore [29]
*N_a_*	Number of astral microtubules from each pole	800	Total number of microtubules per spindle pole ∼300 microtubule “fibers”×average 6 microtubules per fiber [30] – number of kinetochore microtubules above ∼1000
*R_M_*	Radius of microtubule	12.5 nm	[29]
*r*	Radius of particle	5 nm	see **Information S1**
*d_0_*	Critical distance for binding with microtubule	0.8 nm	∼Debye length in physiological saline (see **Information S1**)
*V*	Processive velocity of motorized particle along microtubule	1 µm/s	[12]
*k*	Dissociation rate	1 s^−1^	Processive velocity [12] ÷ processive run length
*D_C_*	Diffusion coefficient in cytoplasm	2 µm^2^/s	Inferred from the dependence of diffusion coefficient on molecular weight and/or size (see **Information S1**)
*D_M_*	Diffusion coefficient along microtubule	0.01 µm^2^/s	[31], [32]
*R_C_*	Radius of cell	10 µm	Chosen by the model in accordance to the normal cell size†
*R_S_*	Radius of spindle	5 µm	Chosen by the model in accordance to the normal mitotic spindle length†

† Variations in these parameters do not affect the qualitative results of the model.

**Figure 1:**
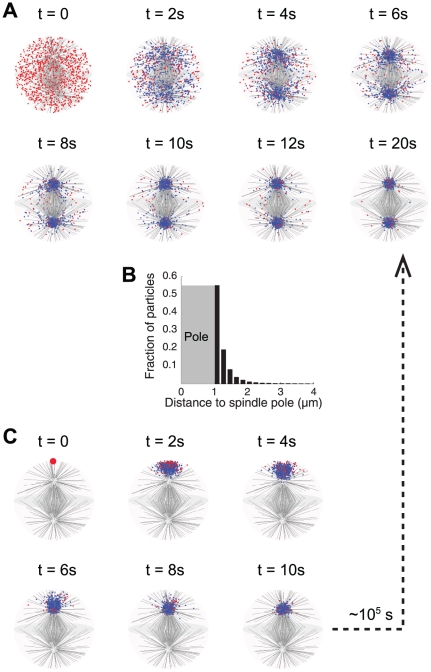
Simulated dynein sequestration by the microtubule spindle. (A) Time series of dynein distribution (also see Movie S1). Dyneins are initially randomly distributed throughout the cytoplasm. Dark grey lines: microtubules (only 1 out of 10 are shown to reduce clutter); blue dots: dyneins bound to microtubules; red dots: dyneins freely diffusing in the cytoplasm. Dyneins become strongly concentrated around the spindle poles within 20 s. (B) Histogram (right) of the equilibrium distribution of dyneins. More than 50% dyneins are concentrated within 200 nm around the spindle pole, and 98% within 1 µm. (C) Dyneins are initially released from one end of the cell, close to one spindle pole (big red dot) (also see Movie S2). Legends are same as in (A). Dyneins become strongly concentrated around the proximal spindle pole within 10 s, but almost none on the opposite spindle pole. Over the simulated duration of 3000 s, only 0.3% dyneins turn over to the opposite pole. Assume that the turnover is a Poisson process, then *prob*(wait time<t) = 1−exp(−*t*/*τ*), where *τ* is the characteristic turnover time, or the reciprocal of the turnover rate. Plugging in *prob* = 0.003 and *t* = 3000 s gives *τ* = 10^5^ s. Both simulations in (A) and (C) are carried out with 1000 non-interacting dyneins and each half-spindle consisting of 800 spindle microtubules and 800 astral microtubules. The dyneins are expelled from the spindle pole immediately as they arrive, i.e. the spindle poles are reflecting boundaries.

We started with the simulation of a 3D mitotic spindle-dynein system. Located in a cell of 20 µm in diameter, the model spindle consists of microtubules originated from two opposite spindle poles distanced by 10 µm. Each spindle pole organizes 800 microtubules within the 90°-cone, which is centered along the spindle axis and based at the mid-plane. Each pole also organizes 800 astral microtubules outside the cone (**Figure 1**). The density of microtubules is essentially 6 times higher within the spindle cone because the cone encases ∼15% of the surface area on a spherical surface. Each microtubule is represented by a cylinder of 25 nm in diameter, extending from the spindle pole to the mid-cell plane or the cell boundary.

### Sequestration by mitotic spindle

Motivated by the observed dynein-mediated sequestration of checkpoint proteins [1], [2], and germ plasm components at the spindle poles [4], we first used our computational model to simulate the spatial regulation of dyneins by the mitotic spindle, and the conclusion naturally extends to the cargoes of the dyneins. Our simulation demonstrated that dyneins are indeed intensely sequestered by the microtubule spindle, and highly concentrated around the spindle poles (**Figure 1**). The results directly explain the observations on dynein localization by Lerit *et al* [4] mentioned in the Introduction. If dyneins are initially evenly distributed throughout the cell, within 20 s they concentrate onto the two spindle poles (**Figure 1**A and Movie S1). At equilibrium, 98% dyneins are located within 1 µm around the spindle poles (**Figure 1**B). This scenario corresponds to the experimentally observed spindles that are sufficiently immersed in the cortex layer; the germ plasm components originally in the cortex are concentrated onto the spindle poles (right of fig.6B and right of fig.3C in [4]). For the few observed spindles that only touch the cortex layer with one pole (middle left of fig.6B and lower left of fig.3C in [4]), we simulated the transport process with the dyneins initially released from one end of the cell, close to one of the spindle poles. Within 10 seconds, the dyneins are strongly concentrated on the proximal spindle pole, but almost none on the opposite pole (**Figure 1**C and Movie S2). Although dyneins will eventually equilibrate to an equal partition between the two spindle poles, like the equilibrium state resulting from the symmetric initial distribution, the time scale for the turnover is on the order of tens of hours (∼10^5^ s), much longer than the relevant time scale for germ cell formation, or even the life span of the syncytial embryo (∼3 hrs). Thus, the model suggests that the microtubule-mediated sequestration effect can last long enough and serve as a robust mechanism to govern the spatial localization of signaling proteins in cellular processes. In this case, the two halves of the microtubule spindle essentially partition the cytoplasm into two non-interacting regions for the dynein-associated molecules.

**Figure 2:**
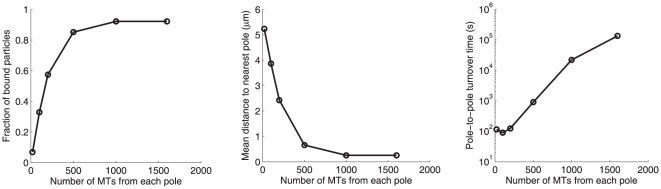
Effects of microtubule density on the spatial regulation. The sequestration (fraction of particles bound), concentration (inverse of mean distance to MTOC) and pole-to-pole turnover time increases, as microtubule density increases.

Intuitively, the microtubule density is critical for the spatial regulation effect; thus the change in the microtubule density over the cell cycle can affect the spatial pattern of the microtubule-binding molecules. **Figure 2** demonstrates that the increase of microtubule density enhances the sequestration effect (defined as the fraction of bound particles) and the concentration effect (defined as the inverse of the mean distance of the particles to the spindle poles), and elongates the pole-to-pole turnover time. Sharp changes occur between 100 and 1000 microtubules per pole/MTOC. This is right about the range over which the microtubule density is regulated through the cell cycle. During mitosis, each spindle pole organizes around thousand microtubules, while an interphase centrosome organizes around hundred microtubules. Therefore, by changing the density of the microtubule network, the cell is able to achieve differential regulatory effects on the microtubule-binding molecules.

### Asymmetric partitioning by asymmetric spindle

**Figure 3:**
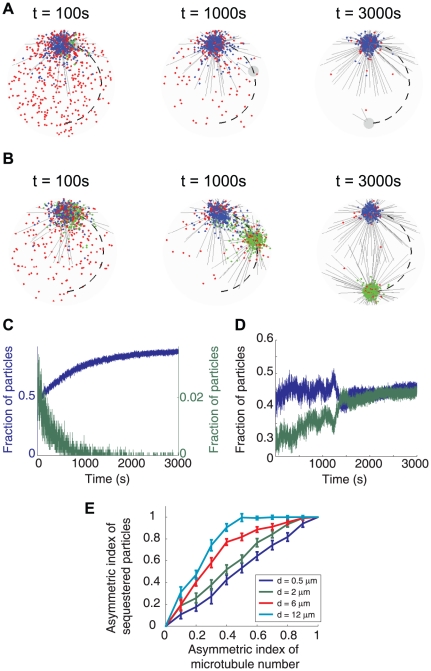
Delayed centrosome maturation causes asymmetric particle partitioning. (A) Time series of dynein distribution with ∼1 hr time delay between the centrosome maturation (also see Movie S3). During the simulated duration of 3000 s, the number of microtubules organized by the dominant centrosome increases from 200 to 800. The second centrosome organizes 20 microtubules throughout the time. It migrates along the trajectory depicted by the dashed curve. Dyneins are sequestered by the dominant centrosome. Dark grey lines: microtubules (only 1 out of 10 are shown to reduce clutter); blue dots: dyneins bound to the microtubules organized by the dominant centrosome; green dots: dyneins bound to the microtubules organized by the second centrosome; red dots: dyneins freely diffusing in the cytoplasm; grey circles: centrosomal area. (B) Time series of dynein distribution with ∼5 min time delay between the centrosome maturation (also see Movie S4). During the simulated duration of 3000 s, the number of microtubules organized by the first centrosome increases from 200 to 800; the number of microtubules organized by the second centrosome increases with the same rate from 150 to 750. The second centrosome migrates along the same path as in (A) (dashed curve). Dyneins ends up equally distributed around the two centrosomes. Legends are same as in (A). (C) and (D) Time trajectories of the number of dyneins bound to the microtubules associated with each centrosome. (C) corresponds to the case in (A), and (D) corresponds to (B). Blue line: the centrosome that matures earlier; green line: the centrosome that matures later (right axis in (C)). With long time delay in centrosome maturation, the number of dyneins sequestered by the second centrosome decreases to nearly zero before the centrosome matures. With short time delay, the original bias in the partitioning of dyneins diminishes as the relative difference in the numbers of microtubule organized by the two centrosomes reduces. (E) Asymmetry of particle partitioning depends on the asymmetry of microtubule numbers organized by the two centrosomes, as well as the distance between the centrosomes. The asymmetric index is defined as (N_1_−N_2_)/(N_1_+N_2_), where N_1_ and N_2_ are the corresponding steady state particle numbers or microtubule numbers associated with each centrosome. The simulations were run with a total number of 1000 microtubules partitioned differentially between the two centrosomes.

Because the microtubule density is critical for the sequestration effect, an asymmetrically organized microtubule spindle would lead to asymmetric partitioning of the molecules. This can serve as a possible mechanism for the asymmetric cell division if the cell fate determinants are spatially regulated by the microtubule network. For example, the *Drosophila* central brain neuroblast divides asymmetrically into a neuroblast and a non-neuroblast daughter. The neuroblast daughter inherits the dominant centrosome, which, during the preceding mitosis, matures and organizes a dense microtubule array ∼1 hr earlier than the other centrosome [7]. We used the computational model to mimic the asymmetric centrosome maturation process and explore the dynein-mediated spatial regulation effect by the asymmetrically growing microtubule network (**Figure 3**A and C, Movie S3). During the 3000 s simulation, the number of microtubules organized by the dominant centrosome increases from 200 to 800, while the second centrosome only organizes 20 microtubules throughout the time. The second centrosome migrates from the vicinity of the dominant centrosome to the opposite side of the cell (dashed curve). As the dominant centrosome matures and grows a denser microtubule array, it imposes increasing sequestration on the dyneins. At the end of the simulation, almost all the particles are concentrated around the dominant centrosome. In the neuroblast cell, the second centrosome starts to mature at this time [7]. As we showed above, it takes ∼10^5^ s for particles to exchange between mature spindle poles (**Figure 1**). The huge kinetic trap prevents the particles from redistributing to the second centrosome within the time scale of mitosis. Moreover, the asymmetric distribution of the cell fate determinants requires a sufficient time delay between the maturation of the microtubule arrays organized by the two centrosomes. For a short time delay between the centrosome maturation or simply small fluctuations in the maturation process, the resultant relative differences in the microtubule density between the two arrays will gradually diminish as the number of microtubules grows. The original biased partitioning of dynein-directed particles is quenched before the microtubule density is high enough to establish the huge kinetic trap that sustains the bias (Figure 3B and D, Movie S4). This simulation showed that the microtubule network is able to achieve both asymmetric and symmetric spatial regulation, simply by controlling the time delay between the maturation of the microtubule arrays organized by each centrosome.

We further predict that asymmetric particle partitioning not only depends on the level of asymmetry between the two microtubule arrays, but also hinges on the distance between the centrosomes (**Figure 3**E). At short inter-centrosome distances, achieving asymmetric particle sequestration requires a much higher level of asymmetry in the microtubule density than at large inter-centrosome distances. Our results thus suggest that the observed asymmetric sequestration effect [7], [10] could also be regulated by the inter-centrosome distance. Experiments that manipulate the microtubule density organized by each centrosome (such as by interfering the γ-tubulin recruitment level) and the inter-centrosome distance shall be a good test for these model predictions (Figure 3E).

### Sequestration pattern in syncytial embryo

So far we have discussed the spatial regulation of dynein-directed particles. The concentration effect evidently relies on the minus-end motility along the microtubules. But the sequestration by microtubules also works without dynein-mediated active transport. Simple binding with microtubules is enough to generate a significant sequestration effect that affects the spatial distribution and dynamics of the molecules. In fact, such sequestration effects apply to the actin-binding molecules and the actin network, too. Therefore, the following case sheds light on the general cytoskeleton-mediated sequestration, although we adopt the parameters for the microtubule system.

**Figure 4:**
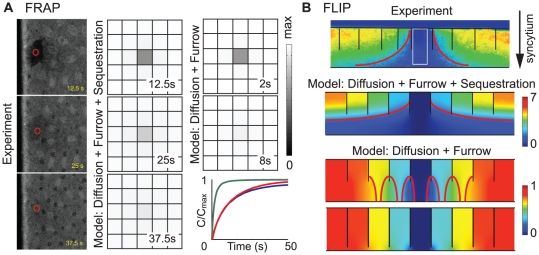
Restricted diffusion of Dorsal in the syncytial embryo. For the sake of computational efficiency on the much larger spatial domain, the simulation was carried out using field equations that depict the density profile of the Dorsal protein, instead of agent-based method that traces each protein particles (see Methods). The field equations were defined on a rectangular block of dimensions 35 µm W×35 µm D×14 µm H. The top area of 7 µm in height are divided into 5×5 cubic subdomains with impermeable vertical boundaries between each other. These subdomains represent the nuclei-associated energids in the syncytial embryo cortex. There are no boundaries between each subdomain and the rest of the space. (A) Comparison with FRAP experiment, top view (also see Movie S5, S6). Left column: experiment data. Middle column: computational result with microtubule-mediated sequestration and furrows. Top two panels in the right column: computational result with pure diffusion restricted by furrows only. The gray scale bar shows the normalized concentration with respect to the maximum concentration at time 0 in the computational results. Bottom panel in the right column: temporal curves of Dorsal concentration at the central point of the photobleached domain, with sequestration (blue), with pure diffusion at *D* = 20 µm^2^/s (green), or with pure diffusion at *D* = 3 µm^2^/s (red). The sequestered case thus demonstrates a phenomenological diffusion coefficient of ∼3 µm^2^/s. (B) Comparison with FLIP experiment, side view (also see Movie S7, S8, S9). Top: experiment data at t = 108 s. Middle: computed concentration profile with microtubule-mediated sequestration and furrows at t = 108 s. Bottom: computed concentration profile with pure diffusion restricted by furrows only at t = 108 s, with the gap width between neighboring energids of 7 µm (upper) or 1 µm (lower). The black lines mark the membrane boundaries between neighboring energids. The white box shows the area of photobleaching. The red lines show the characteristic shape of the contour lines of the concentration profiles. The color bar shows the relative Dorsal concentration. In both the FRAP and FLIP simulations, the effective binding rate *k_on_* = 5 s^−1^ (∼microtubule density of 3 µm^−2^, or 150 microtubules within each energid, cf. Figure S1), the unbinding rate *k_off_* = 1 s^−1^. The diffusion coefficient of unbound protein *D* = 20 µm^2^/s except for the red temporal curve in (A) (cf. [26], the cytoplasmic diffusion coefficient of GFP is 25 µm^2^/s and the molecular weight of Dorsal (∼90 kD, [27]) is about 3 times that of GFP (∼30 kD, [28])). The experimental data are adapted from Delotto *et al*, 2007 [13].

The cytoskeleton, including the microtubule network and the actin network, turns the cytoplasmic space into a so-called “structured cytoplasm”, a concept proposed years ago [14], [15]. The “structured cytoplasm” can partially hold the cytoskeleton-binding components. For example, in a couple of cell cycles before the cellularization of the syncytial *Drosophila* embryo, Dorsal proteins are distributed in the cortex of the embryo (fig.5F of [13], also see **Figure 4**A, left column), where the nuclei-associated energids are semi-separated by the plasma membrane furrows and enriched with cytoskeleton [16], [17]. Fluorescence recovery after photobleaching (FRAP) and fluorescence loss in photobleaching (FLIP) experiments showed delayed exchange of Dorsal proteins between neighboring energids (fig. 5E and 5F of [13], also see **Figure 4**B, top row).

Here we used our computational model to show that the microtubule-mediated sequestration can help explain the observed delayed diffusion. Since the interphase microtubule network is usually extensive throughout the cell, distributed with fairly even density, we simplified our simulation, using field equations to describe the spatial concentrations of the bound and unbound Dorsal proteins. The binding was characterized by an effective binding rate derived from the agent-based simulation (Figure S1).

For the FRAP experiment, the computational results showed that pure diffusion restricted by furrows only evens out the concentration difference between the bleached and the unbleached energids in <10 s (**Figure 4**A and Movie S6). But with the microtubule-mediated sequestration, the FRAP takes ∼40 s as observed in the experiment (**Figure 4**A and Movie S5). The sequestration-hindered diffusion gives a phenomenological diffusion coefficient ∼3 µm^2^/s (**Figure 4**A, lower right panel), much lower than the actual cytoplasmic diffusion coefficient of the free particles, *D* = 20 µm^2^/s. The effective binding rate used in the simulation, *k_on_* = 5 s^−1^, corresponds to the microtubule density of 3 µm^−2^, or 150 microtubules per energid. This is a reasonable number for interphase cells.

Our model also recapitulated the observation from the FLIP experiment, in particular, the characteristic horn-shaped contour lines from side view (**Figure 4**B and Movie S7). These contour lines result from the superposition of the xy-gradient (parallel to the cortex) due to the photobleaching and the z-gradient (perpendicular to the cortex) due to the sequestration. Without the sequestration effect, the membrane furrows alone can also slow down the effective diffusion (Daniels *et. al.*, unpublished result). But these geometric obstacles alone cannot reproduce the observed z-gradient (Figure 4B and Movie S8, S9), i.e., the increasing concentration gradient from the deeper syncytium towards the embryo cortex. Instead, it leads to contour lines that resemble flipped bowls, even if the gaps between energids are very small (Figure 4B). These bowl-shaped contour lines reflect the diffusional fluxes of Dorsal through the gaps. In fact, the z-gradient exists even in the steady state before FLIP (fig.5F of [13]), which, according to our model, is a telltale sign for the sequestration by the “structured cytoplasm” of the energids.

## Discussion

Our agent-based stochastic simulation suggests that the microtubule-mediated sequestration effect can strongly affect the spatial localization of the molecules that directly or indirectly associate with microtubules. Conceptually, a dense microtubule network, along with its associated molecules, can be considered as a subcellular compartment in the cell, even though they are not enclosed by plasma membrane. This is in contrast to the widely accepted membrane-bounded spatial regulation mechanism. We suggest that the microtubule-based sequestration provides a general and robust mechanism that regulates the spatial localizations of cellular components (e.g. proteins and vesicles). In particular, the spatial localization effect is modulated in a cell cycle dependent manner, because the microtubule network, especially the density of microtubules, undergoes significant changes through the cell cycle. The application of the model on asymmetric cell division also sheds important light on stem cell biology.

We should note that, our model is reminiscent of the previously speculated concept of “spindle matrix” [18]. The microtubule spindle associated proteins, e.g. Lamin B and NUMA, haven been posited to tether non-microtubule material around the spindle over a large area, thus “compartmentalizing” many key proteins and membranous structures during mitosis. These additional tethering factors could enhance the effective microtubule-binding affinity, further potentiating the microtubule-mediated sequestration effect demonstrated by our model. Although we focused mainly on microtubules in this work, the same principle applies to the actin cytoskeleton, too. The actin-based network could provide similar sequestration effects of the particles with similar binding-unbinding kinetics.

Our model on dynein-mediated spatial regulation naturally extends to how kinesins, which travel to the opposite direction along the microtubules, regulate the spatial distribution of their associated cargoes. This issue was previously examined by another agent-based model [19]. Simulation with our model showed similar results as in [19]. Microtubule asters sequester kinesins to a much weaker extent than they do dyneins: there is only 2∼3 times increase in the number of kinesins accumulated in the equator zone (1 µm from the equator plane) as compared to the case without microtubule sequestration (**Information S3**). The weaker accumulation of kinesins at the cell equator is due to the astral geometry of the microtubule arrays. As the kinesin travels away from the MTOC, the microtubule array becomes sparser. Once the kinesin falls off from the microtubule, the sparser microtubule array allows much more room for diffusing and lesser chance for re-binding, effectively reducing the phenomenological binding rate of the kinesin to the microtubule array. Our model also suggested a moderate spatial regulation effect by the topology of the microtubule spindle. The sequestration of kinesins at the cell equator is moderately improved if the microtubule asters from the spindle poles are connected by barrel-shaped, anti-parallel microtubule arrays at the middle of the spindle (**Information S3**). Such spindle architecture was suggested by some previous experiments [20].

The spatial localization effect of the microtubule-dependent mechanism is also regulated by other factors, e.g. binding affinity between the particle and the microtubule (Figure S3), the length of microtubules (Figure S4), the association and dissociation with motor proteins, etc. For example, the endosomal vesicles concentrate around the centrosome/MTOC during the interphase in a dynein-dependent manner [21]. This significant concentration around the MTOC at low microtubule densities suggests that the binding affinity between the endosomal vesicles and the microtubule is higher than that achieved by a single dynein (significant concentration effect emerges at unbinding rate <0.1 s^−1^ for a density of 200 microtubules in the whole cell, according to Figure S3). This could possibly result from the association with multiple dyneins and the consequent prolonged binding to microtubules. Meanwhile, endosomal vesicles at different stages (i.e. early endosome, late endosome, trans-Golgi, etc.) are localized differentially through the same microtubule network [22]. Similarly, in the *Drosophila* oocyte, various mRNA species are recruited at different regions of the cell in a microtubule-dependent manner [23], [24]. These variations in the localization patterns on the same microtubule network can only be achieved by regulating the binding affinity to the microtubule, as well as regulating the association with different types of motor proteins and their motility. All these regulatory factors themselves can change with the cell cycle, enhancing the regulatory effect of the cell cycle dependent changes in the microtubule organization. For instance, when the cell enters mitosis, the inhibition of the exocytosis pathway reduces the surface area of the cell membrane and allows the cell to round up [25]. The endosomal vesicles are essentially sequestered by the microtubule network. Since these vesicles are normally transported both by dyneins and kinesins, their sequestration could result from increased activity of the associated dyneins, decreased activity of the associated kinesins, modulated association with these motors, as well as changes in the microtubule organization and density.

In conclusion, our computational model suggests an unexplored functional role of the microtubule network in regulating the spatial localization of cellular components. The microtubule network could be defined as an organelle that compartmentalizes cytoplasm, limits random diffusion, facilitates directed transportation, and thus causes differential spatial distributions of various cellular components.

## Methods

### Agent-based simulation

The scheme of the agent-based simulation is given in Figure S2. Let 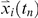 and 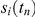 denote the position and the state of the *i*-th particle at the discrete time point *t_n_*. Let 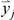, a 3-by-2 matrix represent the position of the minus end and the plus end of the *j*-th microtubule. The minus ends are located at the spindle pole (spherical surface of radius 1 µm). The plus ends are located at the cell cortex or the cell equator, depending on which is reached first. The microtubules do not undergo dynamical changes, i.e. 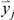 are time invariant. This is because the binding and unbinding largely depends on the local microtubule density, as shown by the results. Then, the equations that govern the agent-based model read as follows, where 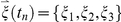 are independent random numbers drawn from the standard normal distribution *N*(0, 1), and 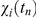 are independent random numbers drawn from the uniform distribution *U*(0, 1).
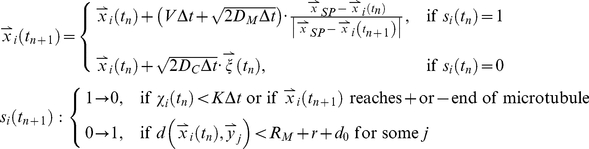
(1)


### Mean field simulation

Let *ρ_f_* be the density of free particles and *ρ_b_* the density of bound particles. They are computed with the following equations,
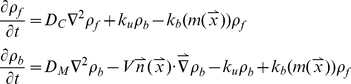
(2)where *D_C_*, *D_M_* are the diffusion coefficients of the particle in the cytoplasm and along the microtubule respectively; *V* is the velocity (≠0 if the particle is driven by molecular motors); 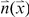 is the unit vector in the direction of the microtubule, pointing toward the corresponding MTOC; *k_u_* and *k_b_* are the unbinding and binding rates of the particle with the microtubule. The binding rate *k_b_* depends on the local microtubule density 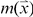. The relation between the binding rate and the microtubule density is given in Figure S1. **Information S2** gives more details of the derivation, as well as a compatibility check with the agent-based model.

## Supporting Information

Figure S1 **Microtubule density vs. effective binding rate to the microtubule network.** 2D agent-based model is simulated to give effective binding rates to microtubule arrays of different densities with different particle sizes (1 nm, 5 nm or 10 nm in radius). In the 2D simulation the microtubule was simplified as a circle of 25 nm in diameter, and placed at the center of a square box with side length equal to the reciprocal of the designated microtubule density. The sides of the square box were set as periodic boundaries, and the microtubule circumference as reflecting boundary for the particle. Like in the 3D simulation, the particle binds to the microtubule when their shortest distance is smaller than *d*_0_, and unbinds with rate *k*. Effective binding rates were inferred from the fraction of bound particles at various microtubule densities (i.e. different box sizes). The effective binding rates only increase by 2 fold for a 10-fold increase in particle size. The curve bends upward at large particle size because volume exclusion effect becomes significant when the particles are crowded by high microtubule density.
(EPS)

Figure S2 **Cartoon scheme of the agent-based simulation.** A free particle diffuses in the cell with a diffusion coefficient, *D*. It binds to a microtubule when it comes within a critical distance, *d*_0_, from the microtubule. It unbinds from the microtubule with a dissociation rate, k. If the particle represents a molecular motor and/or motor-cargo complex, then the particle, when bound, will travel along the microtubule unidirectionally with a velocity, *V*.
(EPS)

Figure S3 **The binding affinity between the particle and the microtubule affect the localization pattern of the dynein-associated particle.** The simulation was carried out on a spherical cell with a single MTOC at the center of the sphere. Dynein-associated particles tend to concentrate around the MTOC. The concentration effect increases in a sigmoidal fashion as the binding affinity between the dynein and the microtubule increases. The critical binding affinity shifts as the microtubule density changes.
(EPS)

Figure S4 **Microtubule length also affects spatial regulation.** (A) 200 5-µm long microtubules around one pole, and 1000 microtubules of full half-cell span around the other. Almost all particles are sequestered by the second half-spindle with denser and longer microtubules. (B) 200 microtubules of full half-cell span around one pole, and 1000 2-µm long microtubules around the other. Only 40% particles are sequestered by the second half-spindle although it organizes more microtubules. Only 1 out of 10 microtubules are shown. While the microtubule density regulates the affinity of dyneins to the microtubule array, the spatial span of the structure determines how soon the particles can find this array by pure diffusion through the microtubule-free space, as well as how easy the particles can escape from the dense, but short microtubule array.
(EPS)

Movie S1 **Simulated dynein sequestration by the microtubule spindle, with all dyneins initially diffusive and homogeneously distributed in the cytoplasm.** Legends follow those in **Figure 1**.
(GIF)

Movie S2 **Simulated dynein sequestration by the microtubule spindle, with dyneins initially released from the vicinity of one pole. Legends follow those in** **Figure 1** **.**
(GIF)

Movie S3 **Delayed centrosome maturation (>1 hr) causes asymmetric partitioning of dyneins.** The number of microtubules organized by the dominant centrosome increases from 200 to 800. The second centrosome organizes 20 microtubules throughout the time. Only 1 out of 10 microtubules are shown.
(GIF)

Movie S4 **Small delay in centrosome maturation (∼5 min) maintains symmetric partitioning of dyneins.** The number of microtubules organized by the first centrosome increases from 200 to 800; the number of microtubules organized by the second centrosome increases with the same rate from 150 to 750. Only 1 out of 10 microtubules are shown.
(GIF)

Movie S5 **Simulated FRAP result of Dorsal distribution in the syncytial embryo, with the influence of microtubule-mediated partial sequestration and the semi-separative furrows.**
(GIF)

Movie S6 **Simulated FRAP result of Dorsal distribution in the syncytial embryo, with pure diffusion restricted by the semi-separative furrows only.**
(GIF)

Movie S7 **Simulated FLIP result of Dorsal distribution in the syncytial embryo, with the influence of microtubule-mediated partial sequestration and the semi-separative furrows.**
(GIF)

Movie S8 **Simulated FLIP result of Dorsal distribution in the syncytial embryo, with pure diffusion restricted by the semi-separative furrows only.**
(GIF)

Movie S9 **Simulated FLIP result of Dorsal distribution in the syncytial embryo, with pure diffusion restricted by the semi-separative furrows only.** The gaps between the neighboring energids narrow down to 1 µm.
(GIF)

Information S1 **Choice of parameters for the agent-based simulation, in particular, the diffusion coefficient, binding distance, time step and particle size.**
(DOC)

Information S2 **Derivation of the mean field model and check of compatibility with the agent-based model.**
(DOC)

Information S3 **Kinesin-mediated spatial regulation by the microtubule network is less intensive than the dynein-mediated regulation.** The effect moderately depends on the architecture of the microtubule network.
(DOCX)

## References

[1] Howell BJ, Hoffman DB, Fang G, Murray AW, Salmon ED (2000). Visualization of Mad2 dynamics at kinetochores, along spindle fibers, and at spindle poles in living cells.. Journal of Cell Biology.

[2] Howell BJ, McEwen BF, Canman JC, Hoffman DB, Farrar EM (2001). Cytoplasmic dynein/dynactin drives kinetochore protein transport to the spindle poles and has a role in mitotic spindle checkpoint inactivation.. J Cell Biol.

[3] Ferrandon D, Elphick L, Nusslein-Volhard C, St Johnston D (1994). Staufen protein associates with the 3′UTR of bicoid mRNA to form particles that move in a microtubule-dependent manner.. Cell.

[4] Lerit DA, Gavis ER (2011). Transport of Germ Plasm on Astral Microtubules Directs Germ Cell Development in Drosophila.. Current Biology.

[5] Rowning BA, Wells J, Wu M, Gerhart JC, Moon RT (1997). Microtubule-mediated transport of organelles and localization of beta-catenin to the future dorsal side of Xenopus eggs.. Proc Natl Acad Sci U S A.

[6] Miller JR, Rowning BA, Larabell CA, Yang-Snyder JA, Bates RL (1999). Establishment of the dorsal-ventral axis in Xenopus embryos coincides with the dorsal enrichment of dishevelled that is dependent on cortical rotation.. J Cell Biol.

[7] Rusan NM, Peifer M (2007). A role for a novel centrosome cycle in asymmetric cell division.. J Cell Biol.

[8] Prehoda KE, Nipper RW, Siller KH, Smith NR, Doe CQ (2007). G alpha i generates multiple Pins activation states to link cortical polarity and spindle orientation in Drosophila neuroblasts.. Proceedings of the National Academy of Sciences of the United States of America.

[9] Siegrist SE, Doe CQ (2005). Microtubule-induced Pins/G alpha i cortical polarity in Drosophila neuroblasts.. Cell.

[10] Gonzalez C, Januschke J (2010). The interphase microtubule aster is a determinant of asymmetric division orientation in Drosophila neuroblasts.. Journal of Cell Biology.

[11] Knoblich JA (2008). Mechanisms of asymmetric stem cell division.. Cell.

[12] King SJ, Schroer TA (2000). Dynactin increases the processivity of the cytoplasmic dynein motor.. Nat Cell Biol.

[13] DeLotto R, DeLotto Y, Steward R, Lippincott-Schwartz J (2007). Nucleocytoplasmic shuttling mediates the dynamic maintenance of nuclear Dorsal levels during Drosophila embryogenesis.. Development.

[14] Karr TL, Alberts BM (1986). Organization of the cytoskeleton in early Drosophila embryos.. J Cell Biol.

[15] Foe VE, Alberts BM (1983). Studies of nuclear and cytoplasmic behaviour during the five mitotic cycles that precede gastrulation in Drosophila embryogenesis.. J Cell Sci.

[16] Mavrakis M, Rikhy R, Lippincott-Schwartz J (2009). Plasma membrane polarity and compartmentalization are established before cellularization in the fly embryo.. Dev Cell.

[17] Frescas D, Mavrakis M, Lorenz H, Delotto R, Lippincott-Schwartz J (2006). The secretory membrane system in the Drosophila syncytial blastoderm embryo exists as functionally compartmentalized units around individual nuclei.. J Cell Biol.

[18] Zheng Y (2010). A membranous spindle matrix orchestrates cell division.. Nat Rev Mol Cell Biol.

[19] Odell GM, Foe VE (2008). An agent-based model contrasts opposite effects of dynamic and stable microtubules on cleavage furrow positioning.. The Journal of cell biology.

[20] Yang G, Cameron LA, Maddox PS, Salmon ED, Danuser G (2008). Regional variation of microtubule flux reveals microtubule organization in the metaphase meiotic spindle.. Journal of Cell Biology.

[21] Montagnac G, Echard A, Chavrier P (2008). Endocytic traffic in animal cell cytokinesis.. Curr Opin Cell Biol.

[22] Dunster K, Toh BH, Sentry JW (2002). Early endosomes, late endosomes, and lysosomes display distinct partitioning strategies of inheritance with similarities to Golgi-derived membranes.. Eur J Cell Biol.

[23] Becalska AN, Gavis ER (2009). Lighting up mRNA localization in Drosophila oogenesis.. Development.

[24] St Johnston D (2005). Moving messages: The intracellular localization of mRNAs.. Nature Reviews Molecular Cell Biology.

[25] Boucrot E, Kirchhausen T (2007). Endosomal recycling controls plasma membrane area during mitosis.. Proceedings of the National Academy of Sciences of the United States of America.

[26] Lippincott-Schwartz J, Snapp E, Kenworthy A (2001). Studying protein dynamics in living cells.. Nat Rev Mol Cell Biol.

[27] Gillespie SK, Wasserman SA (1994). Dorsal, a Drosophila Rel-like protein, is phosphorylated upon activation of the transmembrane protein Toll.. Mol Cell Biol.

[28] Prendergast FG, Mann KG (1978). Chemical and physical properties of aequorin and the green fluorescent protein isolated from Aequorea forskalea.. Biochemistry.

[29] Alberts B, Johnson A, Lewis J, Raff M, Roberts K (2008).

[30] Nedelec F, Kozlowski C, Srayko M (2007). Cortical microtubule contacts position the spindle in C. elegans embryos.. Cell.

[31] Wang ZH, Sheetz MP (1999). One-dimensional diffusion on microtubules of particles coated with cytoplasmic dynein an immunoglobulins.. Cell Structure and Function.

[32] Ross JL, Wallace K, Shuman H, Goldman YE, Holzbaur ELF (2006). Processive bidirectional motion of dynein-dynactin complexes in vitro.. Nature Cell Biology.

