# Supplementary figures and images for: Intracellular Spatial Localization Regulated by the Microtubule Network
Source: PLoS One. 2012 Apr 19;7(4):e34919. doi: 10.1371/journal.pone.0034919 (PMC3330817; doi:10.1371/journal.pone.0034919)

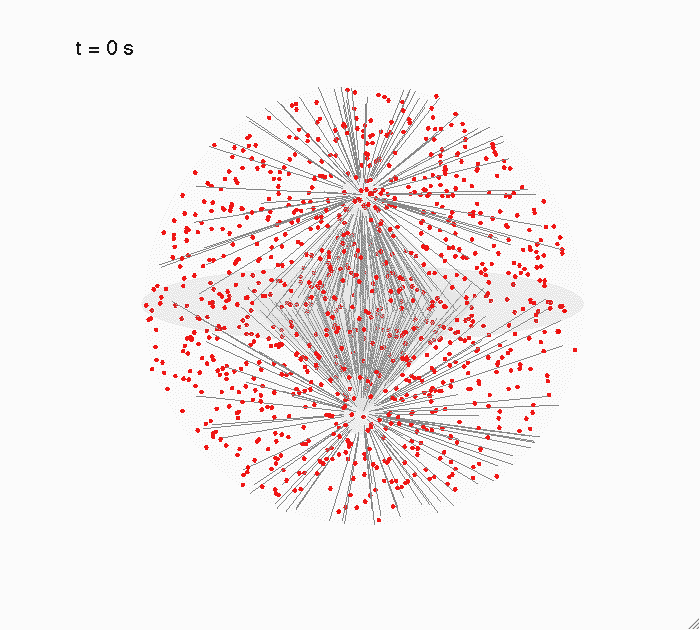

Supplement: Movie S1 — Simulated dynein sequestration by the microtubule spindle, with all dyneins initially diffusive and homogeneously distributed in the cytoplasm. Legends follow those in Figure 1 . (GIF) [file pone.0034919.s005.gif]

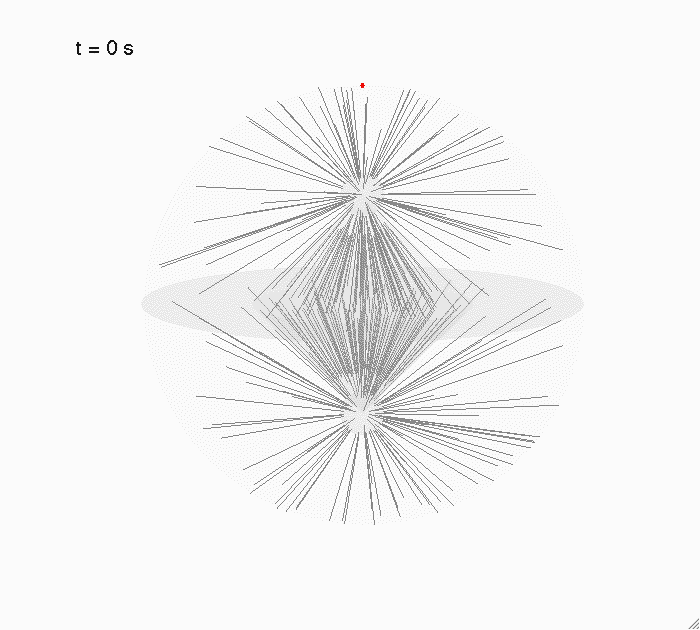

Supplement: Movie S2 — Simulated dynein sequestration by the microtubule spindle, with dyneins initially released from the vicinity of one pole. Legends follow those in Figure 1 . (GIF) [file pone.0034919.s006.gif]

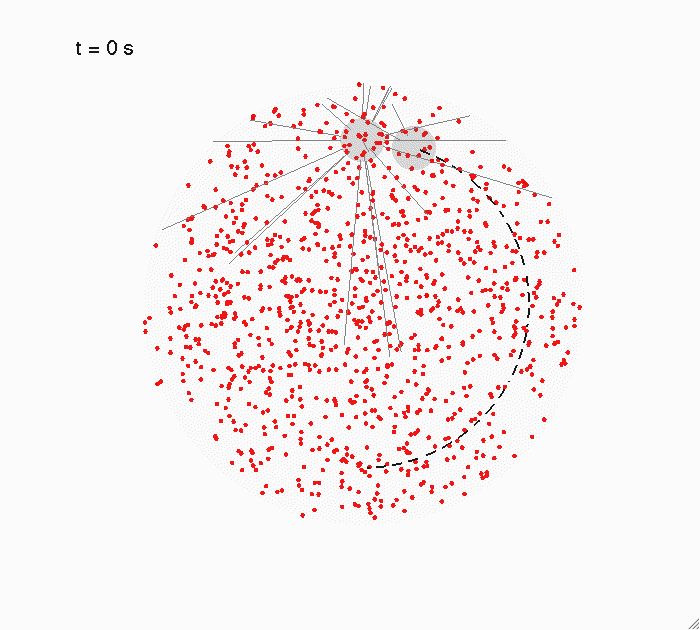

Supplement: Movie S3 — Delayed centrosome maturation (>1 hr) causes asymmetric partitioning of dyneins. The number of microtubules organized by the dominant centrosome increases from 200 to 800. The second centrosome organizes 20 microtubules throughout the time. Only 1 out of 10 microtubules are shown. (GIF) [file pone.0034919.s007.gif]

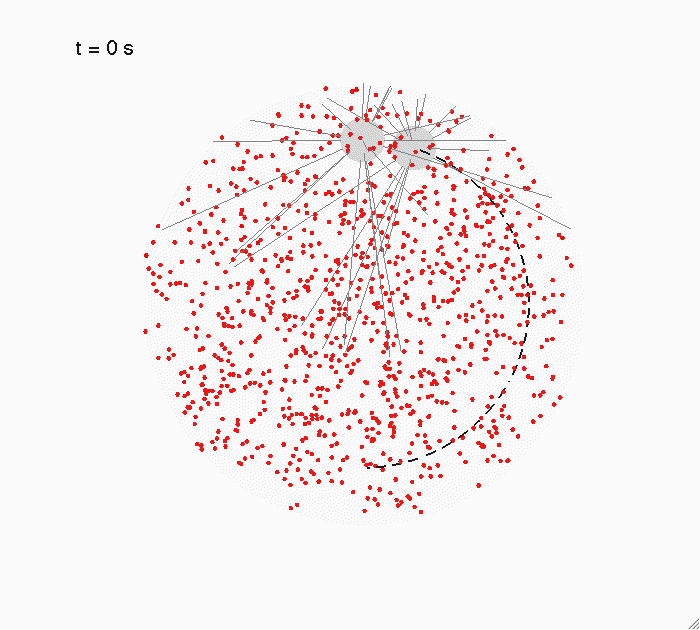

Supplement: Movie S4 — Small delay in centrosome maturation (∼5 min) maintains symmetric partitioning of dyneins. The number of microtubules organized by the first centrosome increases from 200 to 800; the number of microtubules organized by the second centrosome increases with the same rate from 150 to 750. Only 1 out of 10 microtubules are shown. (GIF) [file pone.0034919.s008.gif]

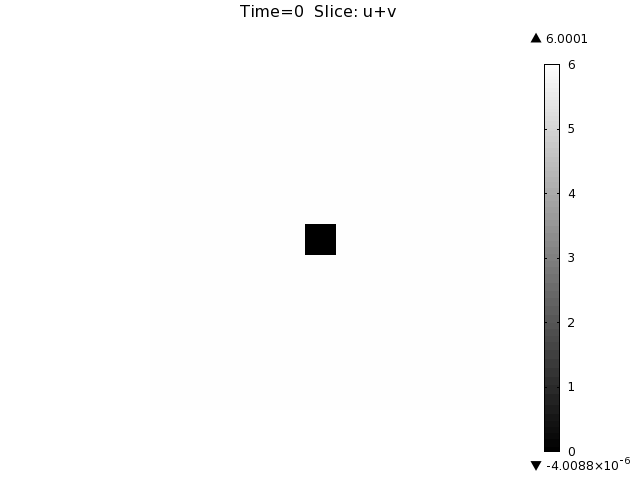

Supplement: Movie S5 — Simulated FRAP result of Dorsal distribution in the syncytial embryo, with the influence of microtubule-mediated partial sequestration and the semi-separative furrows. (GIF) [file pone.0034919.s009.gif]

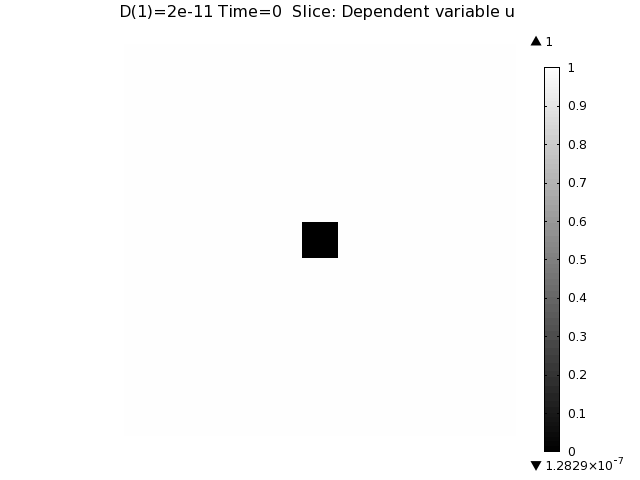

Supplement: Movie S6 — Simulated FRAP result of Dorsal distribution in the syncytial embryo, with pure diffusion restricted by the semi-separative furrows only. (GIF) [file pone.0034919.s010.gif]

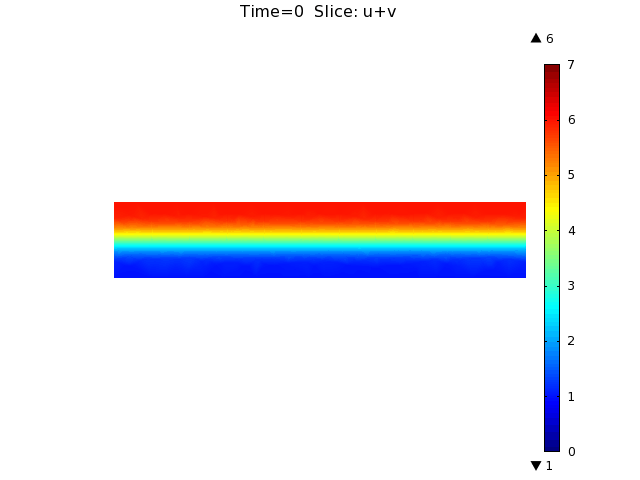

Supplement: Movie S7 — Simulated FLIP result of Dorsal distribution in the syncytial embryo, with the influence of microtubule-mediated partial sequestration and the semi-separative furrows. (GIF) [file pone.0034919.s011.gif]

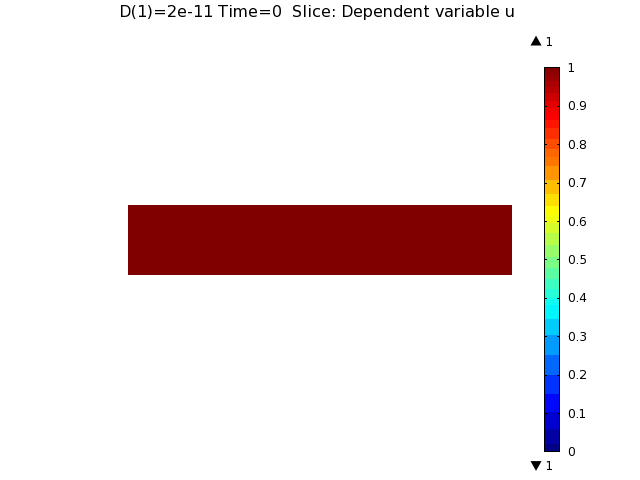

Supplement: Movie S8 — Simulated FLIP result of Dorsal distribution in the syncytial embryo, with pure diffusion restricted by the semi-separative furrows only. (GIF) [file pone.0034919.s012.gif]

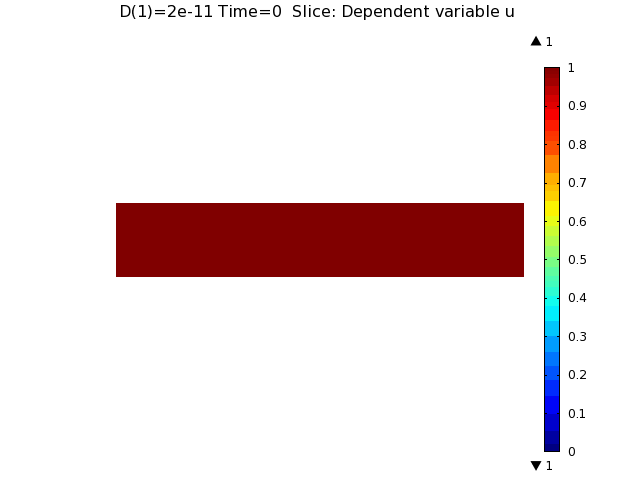

Supplement: Movie S9 — Simulated FLIP result of Dorsal distribution in the syncytial embryo, with pure diffusion restricted by the semi-separative furrows only. The gaps between the neighboring energids narrow down to 1 µm. (GIF) [file pone.0034919.s013.gif]
