# Supplementary material for: Intracellular Spatial Localization Regulated by the Microtubule Network
Source: PLoS One. 2012 Apr 19;7(4):e34919. doi: 10.1371/journal.pone.0034919 (PMC3330817; doi:10.1371/journal.pone.0034919)
Supplement: Information S1 — Choice of parameters for the agent-based simulation, in particular, the diffusion coefficient, binding distance, time step and particle size. (DOC) [file pone.0034919.s014.doc]

## Supporting Information S1

## Choice of parameters for the agent-based simulation

Most of the parameters used in the computer simulation came from experimental measurements/estimates, as given in Table 1 in the main text. There are four parameters that need further elaboration: cytoplasmic diffusion coefficient *D_C_*, binding distance *d*_0_, time step Δ*t*, and particle size *r*.

The results in Figure 1 through Figure 3 of the main text are concerned with the dynein-mediated transport. Yet there is no consensus measurement of the cytoplasmic diffusion coefficient of a dynein. We thus obtained an estimate from a comparison to other molecules with measured cytoplasmic diffusion dynamics. Our estimate was based on two main sources: (1) the measured diffusion coefficient of APC/C^Cdc20^ ~ 1.8 μm^2^/s , and the molecular weight of APC/C^Cdc2^ ~ 1.4 MD is comparable to that of dynein ~ 1.5 MD; (2) the measured diffusion coefficient of cytoplasmic GFP ~ 25 μm^2^/s × (GFP size 3 nm ÷ dynein size 30 nm in length ). Both give diffusion coefficient ~ 2 μm^2^/s. The results in Figure 4 of the main text used a different value to characterize the cytoplasmic diffusion of Dorsal; the reason for that estimate was given in the caption of Figure 4 in the main text.

The binding distance, *d*_0_, is the criterion used in the model to assign the bound state to the particles. When an unbound particle approaches a microtubule by a distance smaller than the binding distance, *d* < *d*_0_, it becomes bound. Note that both the particle and the microtubule have finite size, the distance between the particle and the microtubule is defined as the minimum distance between the two objects, i.e. *d* = vertical distance between the center of the particle and the center line of the microtubule – the radius of the particle – the radius of the microtubule.

In this model, we chose *d*_0_ = 0.8 nm, according to the Debye length of the cytoplasmic environment . In other words, the particle and the microtubule can “see” each other through the electrochemical field when they encounter over such a distance. However, one might ask what if the binding sites of the particle are not aligned towards the microtubule. In fact, the probability of correct alignment in each short-distance encounter is upper-bounded by the ratio of the surface area of the binding site versus the surface area of the whole particle, which is reasonably small. Registering each encounter as binding would cause a serious overcounting of the binding events. But as we will show below, the problem is rescued by choosing an appropriate simulation time step.

The time step, Δ*t*, significantly affects the result of the agent-based simulation. The simulation of the binding to microtubules is essentially a simulation of the first passage time of the particle into a defined target area in the cytoplasmic space. The first passage time tends to be overestimated, and more so when the time step is large. This is because the particle might have reached the target between the discretized time steps, yet these encounters are totally missed by the simulation. The larger the time step, the more encounters missed, resulting in longer first passage time and smaller effective binding rate (Figure S1-1). To avoid this problem, in the conventional computational studies of protein binding, time steps are reduced by orders of magnitude when the particle approaches a defined neighborhood of the target. The reduction of the time step makes the simulation of a few particles very slow, let alone hundreds of particles in our system. Therefore, in our application, we took advantage of the counter-effect between the miss-counting caused by time discretization and the over-counting caused by registering each encounter as binding; we sought an appropriate time step such that the two effects roughly cancels each other out.

**Figure S1-1: The computed binding rate changes with the simulation time step.**

The relaxation time of the rotational fluctuation of the particle serves as a reference for the appropriate time step. The simulated encounters essentially represent all the missed encounters within one time step. Since the particle has sampled through most of the orientations within the relaxation time of the rotational fluctuation, the expected occurrence of correct orientation upon encounters during that time frame should be on the order of 1. The estimated time scale of the rotational fluctuation of a dynein is given in Table S1-1. Our chosen time step, 10^-4^ s, is actually slightly larger. We chose to raise the chance of underestimation against overestimation of the microtubule binding rate, thus keeping our conclusion of the strong microtubule-mediated sequestration effect on the safe side. Table S1-1 also listed a few other important time scales involved in the model. Processes that need to be resolved set the upper bound for the choice of time step, while processes that will not be resolved set the lower bound.

**Table S1-1: Important time scales in the model. Processes that need to be resolved set the upper bound for the choice of time step, while processes that will not be resolved set the lower bound.**

| **Physical process** | **Expression** | **Time scale** | **Type of bound** |
| --- | --- | --- | --- |
| Dynein relaxes from rotational fluctuation | 4π*ηr*^3^/*k_B_*T (*η* is the dynamic viscosity of cytoplasm) | 2x10^-5^ s (20 nm radius) | lower |
| Newly dissociated dynein escapes from the microtubule | *d*^2^/2*D* | 2x10^-7^ s | lower |
| Diffusive dynein encounters any microtubule (i.e. come within *d*_0_) | *ξ*^2^/2*D* (*ξ* is distance between neighboring microtubules) | 2x10^-3^ ~ 0.5 s | upper |
| Dynein unbinds from microtubule | 1/*k* | 1 s | upper |
| Dynein travels to the spindle pole along microtubule | *R_S_*/*V* | 5 s | upper |

For additional check, we compare our estimated microtubule binding rate of the dynein with *in vitro* measurement, ~ 2×10^6^ M^-1^·s^-1^ . 300 microtubules inside a cell of 20 µm in diameter converts to a tubulin dimer concentration ~ 2 mM, leading to a single-molecule binding constant ~ 4 s^-1^. Our simulation gives ~ 10 s^-1^, on the same order.

The size of microtubule-binding molecules varies over a wide range. Here we chose 10 nm in diameter to represent the average size of these molecules. This is smaller than the largest dimension of dynein (~30 nm), and more or less factors in its elongated shape. Besides, decreasing the particle size reduces the effective binding rate. This is shown by 2D simulations with different microtubule densities and particle sizes (Figure S1). Choosing particle size on the lower end thus gives smaller effective binding rate and yields a more conservative sequestration effect. After all, the effective binding rate does not change much with the particle size (Figure S1): it only increases by 2 fold for a 10-fold increase in the particle size.

### Reference

1. Wang ZF, Shah JV, Berns MW, Cleveland DW (2006) In vivo quantitative studies of dynamic intracellular processes using fluorescence correlation spectroscopy. Biophysical Journal 91: 343-351.

2. Lippincott-Schwartz J, Snapp E, Kenworthy A (2001) Studying protein dynamics in living cells. Nat Rev Mol Cell Biol 2: 444-456.

3. Swaminathan R, Hoang CP, Verkman AS (1997) Photobleaching recovery and anisotropy decay of green fluorescent protein GFP-S65T in solution and cells: cytoplasmic viscosity probed by green fluorescent protein translational and rotational diffusion. Biophysical Journal 72: 1900-1907.

4. Ormo M, Cubitt AB, Kallio K, Gross LA, Tsien RY, et al. (1996) Crystal structure of the Aequorea victoria green fluorescent protein. Science 273: 1392-1395.

5. Kikkawa M, Mizuno N, Narita A, Kon T, Sutoh K (2007) Three-dimensional structure of cytoplasmic dynein bound to microtubules. Proceedings of the National Academy of Sciences of the United States of America 104: 20832-20837.

6. Carter AP, Cho C, Jin L, Vale RD (2011) Crystal Structure of the Dynein Motor Domain. Science 331: 1159-1165.

7. Peitzsch RM, Eisenberg M, Sharp KA, Mclaughlin S (1995) Calculations of the Electrostatic Potential Adjacent to Model Phospholipid-Bilayers. Biophysical Journal 68: 729-738.

8. Holzbaur ELF, Johnson KA (1989) Microtubules Accelerate ADP Release by Dynein. Biochemistry 28: 7010-7016.
