# Supplementary material for: Intracellular Spatial Localization Regulated by the Microtubule Network
Source: PLoS One. 2012 Apr 19;7(4):e34919. doi: 10.1371/journal.pone.0034919 (PMC3330817; doi:10.1371/journal.pone.0034919)
Supplement: Information S2 — Derivation of the mean field model and check of compatibility with the agent-based model. (DOC) [file pone.0034919.s015.doc]

## Supporting Information S2

## Derivation of mean field model

The agent-based model corresponds to a mean field model, which describes the spatio-temporal change of the particle density. For complicated boundary geometry like in the example of the Dorsal protein localization in the main text, the mean field model is easier to implement and faster to compute.

We define the density of free particles as *ρ_f_* and the density of bound particles as *ρ_b_*. Their governing equations are:

$\begin{matrix} \frac{\partial\rho_{f}}{\partial t}=D_{C}\nabla^{2}\rho_{f}+k_{u}\rho_{b}-k_{b}(m(\overrightarrow{x}))\rho_{f} \\ \frac{\partial\rho_{b}}{\partial t}=D_{M}\left(\overrightarrow{n}(\overrightarrow{x})\cdot\overrightarrow{\nabla}\right)^{2}\rho_{b}-V\overrightarrow{n}(\overrightarrow{x})\cdot\overrightarrow{\nabla}\rho_{b}-k_{u}\rho_{b}+k_{b}(m(\overrightarrow{x}))\rho_{f} \end{matrix}$ (S1)

In Eq.(S1), *D_C_*, *D_M_* are the diffusion coefficients of the particle in the cytoplasm and along the microtubule respectively; *V* is the velocity (≠ 0 if the particle is driven by molecular motors); $\overrightarrow{n}(\overrightarrow{x})$is the unit vector in the direction of the microtubule, pointing toward the corresponding MTOC; *k_u_* and *k_b_* are the unbinding and binding rates of the particle with the microtubule. The binding rate *k_b_* depends on the local microtubule density $m(\overrightarrow{x})$.

Note that the diffusion of the bound particles is restricted along the microtubule, and thus the Laplacian operator in the second equation of Eq.(S1) only operates along $\overrightarrow{n}(\overrightarrow{x})$. However, in real computation, the lack of second derivatives in the other two dimensions causes numeric instability and spurious oscillations in the result. The problem can be solved by introducing a small diffusion coefficient for the other dimensions. Since *D_M_* is already much smaller than *D_C_*, i.e. the cytoplasmic particles carry out most of the diffusion, we simply applied *D_M_* to all dimensions. The governing equations then convert to:

$\begin{matrix} \frac{\partial\rho_{f}}{\partial t}=D_{C}\nabla^{2}\rho_{f}+k_{u}\rho_{b}-k_{b}(m(\overrightarrow{x}))\rho_{f} \\ \frac{\partial\rho_{b}}{\partial t}=D_{M}\nabla^{2}\rho_{b}-V\overrightarrow{n}(\overrightarrow{x})\cdot\overrightarrow{\nabla}\rho_{b}-k_{u}\rho_{b}+k_{b}(m(\overrightarrow{x}))\rho_{f} \end{matrix}$ (S2)

In the previous section we obtained through 2D simulation the binding rate as a function of the microtubule density (Figure S1). The green line in Figure S1 was used to read off the effective binding rate in the simulation of the localization of Dorsal proteins in the *Drosophila* syncytial embryo (Figure 4 in the main text).

For comparison with the agent-based model, we used the mean field model to compute the density of dynein-associated particles in the mitotic cell, with the same spatially dependent microtubule density. The histogram of the two results compare well (Figure S2-1).

**Figure S2-1: Comparison of the results of the field equation vs the results of the agent-based simulation.** The computation was performed for dynein-associated particles with the full microtubule spindle, i.e. 800 spindle microtubules and 800 astral microtubules per spindle pole. In the field simulation, the microtubule numbers were converted to spatially dependent microtubule density.
