# Supplementary material for: Intracellular Spatial Localization Regulated by the Microtubule Network
Source: PLoS One. 2012 Apr 19;7(4):e34919. doi: 10.1371/journal.pone.0034919 (PMC3330817; doi:10.1371/journal.pone.0034919)
Supplement: Information S3 — Kinesin-mediated spatial regulation by the microtubule network is less intensive than the dynein-mediated regulation. The effect moderately depends on the architecture of the microtubule network. (DOCX) [file pone.0034919.s016.docx]

## Supporting Information S3

## Kinesin-mediated spatial regulation

As discussed in the main text, previous models [[1](#_ENREF_1)] showed that kinesin-mediated transport imposes much weaker sequestration effect than dynein-mediated transport, due to the spread distribution of the plus-ends of the microtubule asters. In order to produce significant equator accumulation, Odell and Foe [[1](#_ENREF_1)] adopted additional mechanisms, including permanently stabilizing part of the microtubules directed towards the equator zone, and maintaining the kinesins at the microtubule tips. These assumptions effectively increase the binding affinity between kinesins and the microtubules in the equator zone, and are therefore able to sequester kinesins in that region.

Here we use our model to investigate the kinesin-mediated spatial regulation. The simulation is same as the one for dynein-mediated spatial regulation, except for switching the velocity of microtubule-bound particles to negative value. All the other parameters remain the same as those given in Table 1 of the main text. Our model showed a moderate accumulation of kinesins around the equator and the cortex of the cell (Figure S3-1A). 31% of total kinesins are accumulated in the equator zone, defined as the cytoplasmic region 1 μm on both side from the equator plane (dashed boxes, Figure S3-1). This region occupies 14.5% of the total cytoplasmic volume. Therefore, kinesin-mediate transport in this case amounts to a ~2 fold accumulation in the equator zone.

Next we investigate the effect of different microtubule architectures on the spatial accumulation. When the two microtubule asters interdigitate with each other, there is only a slight increase in the equator accumulation. But kinesins become more concentrated towards the cortex (Figure S3-1B). Previous experiments suggested barrel-shaped middle spindle, i.e. vertically aligned, anti-parallel microtubules connecting the astral microtubules [[2](#_ENREF_2)]. Simulation with the barrel arrays shows that 45% of kinesins are sequestered at the equator zone, corresponding to a ~3 fold accumulation. Most of the increase is attributed to kinesins sequestered by the barrel arrays themselves (Figure S3-1C). Because of the parallel alignment of the microtubules within the barrel section, kinesins are kept within, instead of being cast outward towards the cortex. Therefore, the architecture of the microtubule spindle imposes a moderate effect on kinesin-mediated spatial regulation.


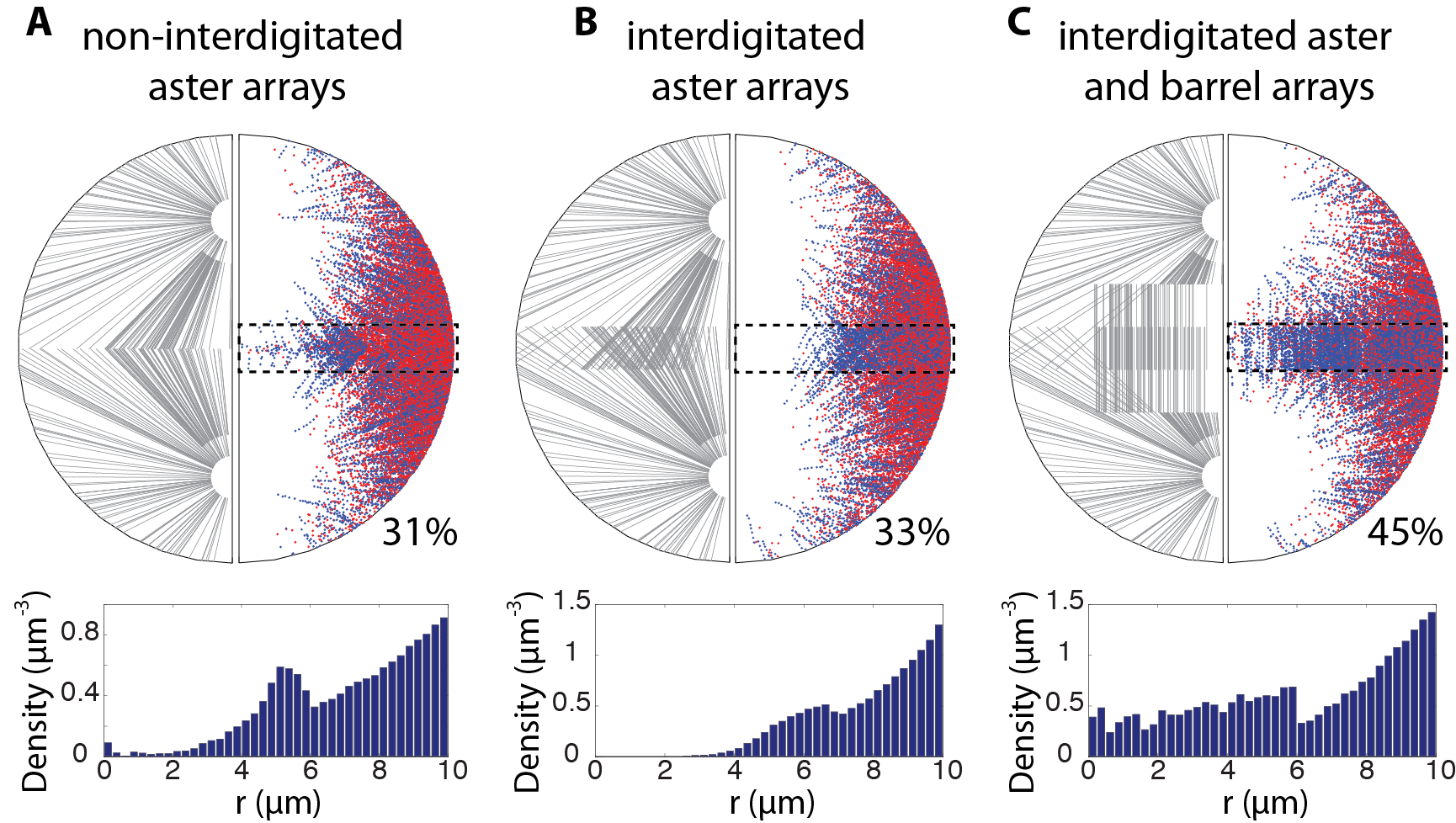


**Figure S3-1: Spatial regulation effect of kinesin-mediated transport.** The upper panels show the structure of the microtubule arrays (only 1 out of 10 microtubules shown) and the resultant distribution of kinesins projected onto the r-z plane (the cell is cylindrically symmetric). The percentage numbers indicate the fraction of kinesins concentrated within the equator zone (dashed boxes). The lower panels show the kinesin densities in the equator zone as functions of the distance to the central axis. (**A**) Two astral arrays without interdigitation. This is the same case as the simulations for dyneins. 31% of total kinesins are distributed in the equator zone (**B**) Two astral arrays interdigitating by 2 μm around the equator zone. The total number of kinesins sequestered by the equator zone only increases mildly compared to (A). But kinesins are more concentrated towards the cell cortex. (**C**) Two interdigitated astral arrays along with two interdigitated barrel arrays. The barrel arrays, each containing 800 microtubules, only span the central area up to 6 μm from the central axis, i.e. only cover the spindle area. They also interdigitate by 2 μm near the equator. In this case, the sequestered kinesins by the equator moderately increase to 45% of total.

The dependence of kinesin distribution on spindle architecture is manifest over a large parameter range. Figure S3-2 gives an example of how the unbinding rate affects the sequestration effect. The difference between the barrel-shaped spindle and the aster-shaped spindle persists as long as the binding affinity between kinesins and microtubules is large enough to sustain considerable equator accumulation.

**Figure S3-2: Effect of spindle architecture on kinesin-mediated spatial regulation.** Interdigitation of the microtubule asters does not significantly improve equator accumulation. But barrel-shaped mid-spindle arrays moderately increase the equator accumulation by better sequestering the kinesins within the anti-parallel microtubule arrays.

### Reference

1. Odell GM, Foe VE (2008) An agent-based model contrasts opposite effects of dynamic and stable microtubules on cleavage furrow positioning. The Journal of cell biology 183: 471-483.

2. Yang G, Cameron LA, Maddox PS, Salmon ED, Danuser G (2008) Regional variation of microtubule flux reveals microtubule organization in the metaphase meiotic spindle. Journal of Cell Biology 182: 631-639.
